# Supplementary material for: Incidence and predictors of anemia among adults on HIV care at South Gondar Zone Public General Hospital Northwest Ethiopia, 2020; retrospective cohort study
Source: PLoS One. 2022 Jan 12;17(1):e0259944. doi: 10.1371/journal.pone.0259944 (PMC8754319; doi:10.1371/journal.pone.0259944)
Supplement: S1 File — (DOCX) [file pone.0259944.s001.docx]

Agimasie Tigabu, BSc, MSc

Lecturer of Adult Health Nursing, Department of Nursing,

College of Medicine and Health Science

Debre Tabor University,

Debre Tabor, ETHIOPIA

Email: [ethiomom23@gmail.com](mailto:ethiomom23@gmail.com)

PLOS ONE staffs

For PLOS ONE staffs, Thank you for giving us the opportunity to confirm the raised question. I am resubmitting this modification November 11, 2021. Our manuscript is PONE-D- 20-26223R1, Incidence and Predictors of Anemia among Adults on HIV care: A Retrospective cohort study.

Thank you once again for your time and attention to our manuscript. We look forward to working with you.

Best regards,

Agimasie Tigabu

**LOS ONE staffs**

1. *Please confirm at this time that the minimal data set supporting the results of your study are within your submission. We hope to hear from you soon.*

Thank you so much for your confirmation request. Yes, this minimal data set is supporting the result of our study.

## Data extraction tool

Questionnaire is prepared for the collection of socio-demographic, socio economic, clinical and treatment-related information on the patients ART medical registration book and patients’ chart to assess incidence and Predictors of anemia among HIV infected adults who are attending HIV care at Debre Tabor General Hospital, Amhara Regional State, Northwest Ethiopia, from 2015 to 2019.

Data collection date-------------------------month-------------------------Year--------------------

Name of the Hospital ----------------------------------------------------------------------------------

Name of data collector--------------------------- signature------------------------------------------

Name of supervisor---------------------------------signature-----------------------------------------

Code No._________________________________

| **Part I: Socio demographic and socio-economic characteristics** | | | **Remark** |
| --- | --- | --- | --- |
| 101 | Date of enrollment to HIV care | ----------/---------/-------- DD/MM/YY |  |
| 102 | Age at enrollment | ----------Year |  |
|  | Current age | ----------year |  |
| 103 | Sex | 1. Male 2. Female |  |
| 104 | Marital status | 1. Single 2. Married 3. Divorced 4. Windowed |  |
| 105 | Residence | 1. Rural 2. Urban |  |
| 106 | Occupation | 1. Not employed 2. Employed |  |
| 107 | Educational status | 1. No education 2. Primary 3. Secondary 4. Tertiary and above |  |
| 108 | Disclosure status | 1. Not disclosed 2. Disclosed |  |
| 109 | Family size | --------- |  |
| 110 | Does the caretaker use any of the substance? | 1. No 2. Yes | If no-goes question No 201 |
| 111 | If yes to question No 110, which of the stated substance used? | 1. Alcohol drinking 2. Cigarette smoking 3. Chewing chat 4. Others(specify) |  |
| **Part II: clinical related characteristics: You can choose more than one, in the multiple-choice.** | | | |
| 201 | WHO clinical staging at baseline | 1. Stage I 2. Stage II 3. Stage III 4. Stage IV |  |
| 202 | CD4 count at base line | --------- cells/μl |  |
| 203 | Viral load at baseline | ----------copies/mL |  |
| 204 | Serum Hemoglobin at baseline | ------------ g/dl |  |
| 205 | Weight | (-------) kg |  |
| 206 | Height | (--------) m |  |
| 207 | BMI in kg/m^2^ | -------- |  |
| 208 | MUAC in cm | ----------- |  |
| 209 | Serum creatinine | ----------mmol/L |  |
| 210 | Serum ALT | ----------U/L |  |
| 211 | Hepatitis B surface antigen | 1. Positive 2. Negative |  |
| 212 | Hepatitis C surface antigen | 1. Positive 2. Negative |  |
| 213 | Functional status | 1. Working 2. Ambulatory 3. Bedridden |  |
| 214 | Past TB history | 1. Yes 2. No |  |
| 215 | If yes to question No 214, what types of TB did the caretaker have? | 1. Pulmonary TB 2. Extrapulmonary TB |  |
| 216 | did the caretaker have past TB Treatment? | 1. Yes 2. No |  |
| 217 | Does the caretaker have comorbid cases | 1. Yes 2. No |  |
| 218 | If yes to Question No 216, what type of comorbidity did the caretaker have? | 1. Intestinal parasite 2. Malaria 3. Asthma 4. COPD 5. allergic diseases 6. Other (specify)------- |  |
| 219 | Opportunistic infection | 1. Yes 2. No |  |
| 220 | If yes, what type of infection | 1. TB 2. CMV 3. PCP 4. Herpes simplex 5. Kaposi sarcoma 6. Toxoplasmosis 7. Encephalopathy 8. Wasting syndrome 9. Herpes zoster 10. PGL 11. PML 12. Candidiasis 13. Diarrhea 14. Pneumonia 15. Other (specify)------- |  |
| **Part III; treatment-related characteristics** | | | |
| 301 | If yes for question No 215, what types of anti-TB treatment regimen had taken | ………….. |  |
| 302 | Date confirmed HIV positive | DD------MM------YY----- |  |
| 303 | Date ART started | DD------MM------YY----- |  |
| 304 | Initial Regimen type | ------ |  |
| 305 | When this regimen was started | - 1. before 2017   2. After 2017 |  |
| 306 | Was the Regimen changed? | 1. Yes 2. No |  |
| 307 | If yes, when it was changed? | DD------MM------YY----- |  |
| 308 | What is the new (changed) regimen? | ------------ |  |
| 309 | Reason for switch | 1. Side effects 2. tuberculosis 3. Others (specify)---------- |  |
| 310 | Duration of treatment | ----------- |  |
| 311 | Level of Adherence | 1. Poor 2. Fair 3. Good |  |
| 312 | If poor or fair Adherence for Question No 311 | In why column note the reason  1.drug side effect  2. share with others  3. forgot  4. felt better  5. too ill  6. stigma, the discloser  7. drug stock out  8. Lost/ran out of pills  9. Delivery/travel problems  10. Alcohol  11. Depression  12. Other(specify)------- |  |
| 313 | OI prophylaxis was given | 1. not given 2. Cotrimoxazole 3. INH  4. Other (specify)-------- |  |
| 314 | Multivitamin supplement | 1.yes 2. No |  |
| **Part IV HIV Care/ART follow up form**   \| Follow-up date (DD/MM/YY) \| \|  \|  \|  \|  \|  \|  \|  \|  \|  \| \| --- \| --- \| --- \| --- \| --- \| --- \| --- \| --- \| --- \| --- \| --- \| \| Months on ART \| \|  \|  \|  \|  \|  \|  \|  \|  \|  \| \| Nutritional assessment \| Wt. (kg) \|  \|  \|  \|  \|  \|  \|  \|  \|  \| \| MUAC \|  \|  \|  \|  \|  \|  \|  \|  \|  \| \| BMI \|  \|  \|  \|  \|  \|  \|  \|  \|  \| \| N, UN/OW \|  \|  \|  \|  \|  \|  \|  \|  \|  \| \| Nutritional supplementation (Y/N) \|  \|  \|  \|  \|  \|  \|  \|  \|  \| \| Functional status (W, A, B) \| \|  \|  \|  \|  \|  \|  \|  \|  \|  \| \| WHO stage (I-IV) \| \|  \|  \|  \|  \|  \|  \|  \|  \|  \| \| TB screen (P/N) \| \|  \|  \|  \|  \|  \|  \|  \|  \|  \| \| TB prophylaxis/Rx \| \|  \|  \|  \|  \|  \|  \|  \|  \|  \| \| OIs \| \|  \|  \|  \|  \|  \|  \|  \|  \|  \| \| CD4 count /mm^3^ \| \|  \|  \|  \|  \|  \|  \|  \|  \|  \| \| Viral load \| \|  \|  \|  \|  \|  \|  \|  \|  \|  \| \| Hemoglobin (Hgb) \| \|  \|  \|  \|  \|  \|  \|  \|  \|  \| \| Alanine aminotransferase (ALT) \| \|  \|  \|  \|  \|  \|  \|  \|  \|  \| \| Aspartate aminotransferase (AST) \| \|  \|  \|  \|  \|  \|  \|  \|  \|  \| \| Cotrimoxazole \| ADH (G, F, P) \|  \|  \|  \|  \|  \|  \|  \|  \|  \| \| Dispense dose \|  \|  \|  \|  \|  \|  \|  \|  \|  \| \| Other medication dispensed \| \|  \|  \|  \|  \|  \|  \|  \|  \|  \| \| ARV drug \| ADH (G, F, P) \|  \|  \|  \|  \|  \|  \|  \|  \|  \| \| Why \|  \|  \|  \|  \|  \|  \|  \|  \|  \| \| Dispense (dose/code) \|  \|  \|  \|  \|  \|  \|  \|  \|  \| \| Side effect \|  \|  \|  \|  \|  \|  \|  \|  \|  \| \| Reason for change \|  \|  \|  \|  \|  \|  \|  \|  \|  \| \| The client set HIV prevention plan \| \|  \|  \|  \|  \|  \|  \|  \|  \|  \| \| Next visit date (dd/mm/yy) \| \|  \|  \|  \|  \|  \|  \|  \|  \|  \| | | | |
| 401 | Follow up | 1. Weekly 2. Monthly 3. Every 3 month 4. >=4 month |  |
|  | Did the patient develop anemia during F/up | 1. Yes, 2. No |  |
| 402 | When was it developed? | / / D D/MM/YY |  |
| 403 | Follow up conclusion | - - - - 1. Alive         2. Dead         3. The loss to follow up         4. Transferred out         5. Other |  |
| 404 | Last visit date | DD____MM____YY______ |  |

Name of data collector -----------------------------sign -----------------------------date ---------------- **Approved by** ------------------------------------------sign -----------------------------date ------------

## Annex 3-WHO HIV clinical staging and level of Adherence criteria

**WHO HIV clinical staging:**

**Clinical stage 1:** a person with confirmed HIV infection who is asymptomatic and/or Persistent generalized lymphadenopathy (PGL)

**Clinical stage 2:** A person with confirmed HIV infection and having:

- Moderate unexplained weight loss (<10% of presumed or measured body weight)
- Recurrent respiratory tract infections (RTIs, sinusitis, bronchitis, otitis media, pharyngitis)
- Herpes zoster
- Angular cheilitis
- Recurrent oral ulcerations
- Papular pruritic eruptions
- Seborrheic dermatitis
- Fungal nail infections of fingers

**Clinical stage 3:** Conditions where a presumptive diagnosis can be made based on clinical signs or simple investigations:

- Severe weight loss (>10% of presumed or measured body weight) Unexplained chronic diarrhea for longer than one month
- Unexplained persistent fever (intermittent or constant for longer than one month)
- Oral candidiasis
- Oral hairy leukoplakia
- Pulmonary tuberculosis (TB) diagnosed in the last two years
- Severe presumed bacterial infections (e.g. pneumonia, empyema, pyomyositis, bone or joint infection, meningitis, bacteremia)
- Acute necrotizing ulcerative stomatitis, gingivitis or periodontitis

**Clinical stage 4:** Conditions where a presumptive diagnosis can be made based on clinical signs or simple investigations:

- HIV wasting syndrome
- Pneumocystis Carinii Pneumonia
- Recurrent severe or radiological bacterial pneumonia
- Chronic herpes simplex infection (orolabial, genital or anorectal of more than one month’s duration)
- Esophageal candidiasis
- Extrapulmonary TB
- Kaposi’s sarcoma
- Central nervous system (CNS) toxoplasmosis
- HIV encephalopathy

**level of Adherence:**

estimate adherence using the table below. Adherence percent missed doses.

| Level of adherence | Percent (%) | (of 30 doses) | (of 60 doses) |
| --- | --- | --- | --- |
| G(good) | $>$95 % | $<2 doses$ | $<3 doses$ |
| F(fair) | 85-94 % | 3-5 doses | 3-9 doses |
| P(poor) | $<85\%$ | $\geq6 doses$ | $>9 doses$ |
