# Supplementary material for: Incidence and predictors of anemia among adults on HIV care at South Gondar Zone Public General Hospital Northwest Ethiopia, 2020; retrospective cohort study
Source: PLoS One. 2022 Jan 12;17(1):e0259944. doi: 10.1371/journal.pone.0259944 (PMC8754319; doi:10.1371/journal.pone.0259944)
Supplement: Attachment — Submitted filename: comments.docx [file pone.0259944.s002.docx]

General comments:

1. This institution-based retrospective cohort study conducted in Ethiopia will add value to the existing literature. In this study, they have found a high incidence rate if anemia, and body mass index, SCr, poor adherence level, and increased ART duration remained significant predictors for developing anemia. However, there is some study bias regarding study population pre-selection which needs to be analyzed and discussed in more detail. Moreover, language polishing is required throughout the text.

Major revisions

Introduction

1. It appeared to be exaggerated. It should be concise and problem-oriented. Please rewrite it and highlight the magnitude and burden of anemia in HIV in the context of Ethiopia and globally as well.
2. Please mention some evidence on mortality and morbidity rate secondary to anemia.
3. The line 58 t0 68 seems exaggerated. Please consider removing these lines.
4. Authors have mentioned the several causes of HIV-related anemia without proper citation of individual independent factors. I would suggest citing each risk factor accordingly. Add vital socio-demographic factors that have been potentially linked to anemia in HIV individuals.
5. Please state the clinical relevance of this study in Ethiopia.

Methods

1. In exclusion criteria, authors have stated only pregnant women and anemia at the beginning of the follow-up as exclusion criteria; however, failed to discuss other potential factors such as active gastric bleeding, and some drugs which may contribute to developing anemia during follow-up.
2. Please highlight the core outcome variables and covariates analyzed in the study.
3. For the sample size calculation, the authors have considered significant predictors for the incidence of anemia from the earlier study. However, it is not clear whether it is the incidence rate or predictors that the authors have considered. Please make it clear.
4. Data collection procedure: Please be consistent with the sentence while writing the conduct of the study. Please consider re-writing it (page no. 134-137).
5. Data were entered using EPI-data Version 3.1. Clear and completed were analyzed using

STATA Version 14 statistical software. The sentence is not clear. Please re-write it.

6. How the missing data were handled?

Results:

7. The median age of the entire cohort was 34 years (IQR; 12). More than half (52.1%)

157 of people living with HIV [PLHIV] were females and 323(78.6%) were Orthodox Christian

158 followed by Muslim 60(14.6%) and protestant 28(6.8%) follower. Majority, 245 (59.6%), of the159 patients came from urban areas. A total of 382 (92.9%) patients had disclosed their HIV status to160 either their husband/wife or other family members.

Please present the above figures in a table format.

8. Clinical and treatment related characteristics should be presented in table form
9. Do the patients have any underlying medical conditions? If yes, please present them in table
10. How about alcohol and smoking habit?
11. The predominant regiments initially prescribed were 174 combination of TDF, 3TC and EFV (1e) 212(51.6%) followed by AZT, 3TC and NVP (1c) 76(18.5%). Please consider changing the word regiments to regimens
12. Two hundred six (60.1%) patients had changed their initial regimen during the follow up period. From the total participants, 26(12.6%) patients switched to second-line HAART.

Some Anti-retroviral agents have a great impact on blood profile which may contribute to anemia. Please mention the type of regimens and its length that were switched to another regimen

13. How did the authors assess the ART adherence level? Please describe it in the methodology section.
14. Authors have described nutritional status based on BMI. I would rather describe BMI as normal, overweight and obese. Please change it.
15. What were the criteria for anemia? Please illustrate this in the methodology section
16. I would suggest highlighting the prevalence rate of anemia at enrollment in the study.
17. Please illustrate the baseline Hb levels of the enrolled patients.
18. SCr was categorized as: SCr <1.1 IU/L and >1IU/L. what are the clinical relevance of this classification.
19. What is the median ART duration? Please present it as a continuous variable as well as categorical ( e.g. <1 year vs >1 year)
20. Drug regimens may have the potential to influence anemia in this particular group of population. Therefore, I would advise authors to consider these analyses in both bivariate and multivariate analysis.
21. I would consider adding P-value for the Bivariate Cox-proportional hazard regression model
22. Please state the variables that were adjusted in the mulvariable Cox regression model.

Discussion

23. This noticeable discrepancy might be related to early initiation of ART for participants in the current study irrespective of CD4 count and WHO clinical staging (**17**) and availability of new drug regimen (dolutegravir containing regimen).

The above statement needs to be justified by the results shown in the study. Authors have failed to demonstrate the proportion of individuals who started early ART. The differences may be due to the availability of dolutegravir containing regimen, which need to be justified by existing literature.

24. Increased serum creatinine related to decrement of renal function to filter it which can result blunt erythropoietin production in response to lower hemoglobin concentration (**30**). This finding suggests that, the need of erythropoietin treatment beside regular monitoring of hemoglobin concentration for patients who have elevated serum

Based on the above statement, it is not clear whether patients have had some kind of renal impairment; and this should be clearly mentioned in inclusion/ exclusion criteria. Moreover, being SCr> 1.1 IU/L as a risk factor for developing anemia does not imply that the patients require erythropoietin treatment. More assessment with respect to renal function is needed.

25. This drug is associated with a more rapid viral suppression and higher genetic resistance when compared with nonnucleoside reverse transcriptase inhibitors.

The above statement seems irrelevant to correlate with the discrepancy seen in the study.

Conclusion

26. Please be focused on major findings and draw the conclusion accordingly. Furthermore, some suggestions seem to be exaggerated. Please consider re-writing it.
