# Supplementary material for: Incidence and predictors of anemia among adults on HIV care at South Gondar Zone Public General Hospital Northwest Ethiopia, 2020; retrospective cohort study
Source: PLoS One. 2022 Jan 12;17(1):e0259944. doi: 10.1371/journal.pone.0259944 (PMC8754319; doi:10.1371/journal.pone.0259944)
Supplement: Attachment — Submitted filename: response to revision comments.docx [file pone.0259944.s003.docx]

Agimasie Tigabu, BSc, MSc

Lecturer of Adult Health Nursing, Department of Nursing, College of Medicine and Health Science

Debre Tabor University, Debre Tabor, ETHIOPIA

Email: [ethiomom23@gmail.com](mailto:ethiomom23@gmail.com)

Anna Fodor,

PLOS ONE

Dear Anna Fodor

My colleagues and I are happy to re-submit to you a revised version of our manuscript PONE-D- 20-26223R1, Incidence and Predictors of Anemia among Adults on HIV care: A Retrospective cohort study. For PLOS One, Thank you for giving us the opportunity to revise and resubmit this manuscript. I am resubmitting this revision on March 28 2021.

We thank you for your detailed and thoughtful suggestions. We have incorporated the recommended changes in the manuscript to the best of our ability and we feel that our revised manuscript is significantly improved as a result of their advice.

We have responded specifically to each suggestion in the memo below and have highlighted our changes in the manuscript text. In the memo, portions of the reviewers’ comments are reproduced in italic text, and our responses to your comments are listed beneath in normal text.

The funders had no role in study design, data collection and analysis, decision to publish, or preparation of the manuscript.

Thank you once again for your time and attention to our manuscript. We look forward to working with you and you to move this manuscript closer to publication.

Best regards,

Agimasie Tigabu

REVIEWER COMMENTS AND RESPONSES

### Editorial Comments

**Anna Fodor:**

1. *We note your Data Availability Statement in the online submission form now reads as follows:

   "The datasets analyzed during the current study are available from the corresponding author upon reasonable request."

   -However, as mentioned, PLOS only permits data to be made available on request if there are ethical or legal restrictions on sharing the data.*

Thank you for your highlight. Even though there are no legal or ethical restrictions on sharing our data publicly, all datasets are available within the manuscript.

Sorry for saying the datasets are available from the corresponding author upon reasonable request from previous online submission form. We have corrected it on the revision submission.
We have also attached the decision of IRB letter previously, and we have written the name of institutional review board committees.
Those committees are; 1. Tadesse Daget,
                                     2. Yinager Workineh
                                     3. Amlaku Mulat
                                     4. Abraham Belachew
                                     5. Chalachaw Genet

2. *Please confirm whether or not this manuscript is under consideration at BMC Infectious Diseases Journal.*
   Thank you for pointing out this comment. We have confirmed that this manuscript is not under consideration at BMC Infectious Diseases Journal
