# Supplementary material for: Incidence and predictors of anemia among adults on HIV care at South Gondar Zone Public General Hospital Northwest Ethiopia, 2020; retrospective cohort study
Source: PLoS One. 2022 Jan 12;17(1):e0259944. doi: 10.1371/journal.pone.0259944 (PMC8754319; doi:10.1371/journal.pone.0259944)
Supplement: Attachment — Submitted filename: Comments.docx [file pone.0259944.s004.docx]

Comments

The authors have attempted to address my previous concerns, yet there are many issues that need to be addressed properly before considering for publication. My specific comments are outlined below:

Abstract

-Anemia is a major public health problem worldwide which accounts 24.8% of the population. Few cross-sectional studies have been conducted on anemia and human immunodeficiency virus. However, it cannot address the incidence and predictors of anemia among human immunodeficiency virus -infected adults.

-The above statement does not support the relevance of the study, as there is a plethora of studies examining this issue globally as well as in Ethiopia also. Please consider rewriting the above statement again

-Moreover, the estimates presented in the background does not seem relevant at this stage

-Methods are not adequately described- such as population selection and outcome measures need to be clearly mentioned.

-The authors stated that a ethical clearance was obtained from the Institutional Review Board of Bahir Dar University, and also, we got permission letter to review charts from the concerned bodies in the hospital

-The above statement should be rephrased

-This is a similar confusion that which factors were from bivariate and multivariate analyses, and need to be very clearly described, and conclusion must be drawn from the appropriate findings.

Background:

-Line 63-67 seems irrelevant and exaggerated. It should be concise and problem-oriented.

-Please highlight the current burden of the disease globally, and especially in Ethiopia

-The authors failed to clearly summarize the current scenario of the topic. There seems to address the limitation of the current knowledge in this field, especially in Ethiopia

-The authors have revealed some data on the prevalence of anemia in Ethiopia; however, in this particular issue, providing data on the incidence of anemia in this particular setting is vital

-Line 87-89

The authors have indicated some contributing factors of developing anemia, yet there are many potential factors that contribute to this burden that needs to be considered.

-A strong rationale behind this study in this setting is needed

-Overall, the introduction needs to be fine-tuned to be more clear. The numerous grammatical errors and language needs to be considered.

Methods:

- In the exclusion criteria, authors have excluded only the pregnant women and anemia at the beginning of the follow-up . How about the other potential factors that may contribute to developing anemia during follow-up?

- Please highlight the core outcome variables and covariates analyzed in the study.

- Authors have assessed the adherence level based on the number of doses. I wonder how did the authors approach this, as the review was retrospective. Moreover, is this a standard tool for the assessment of the adherence level. Need citation and reliability of this tool.

- For the sample size calculation, the authors have used the significant predictors from previous studies. I wonder that there are numerous significant predictors for developing anemia, and which of them the author employed for calculating sample size.

-How the missing data were handled?

-In the methodology section, it is vital to include the clinical criteria for the diagnosis of anemia and its severity.

Results

-data should be presented in a well-fashioned manner. I see there are many results described which were not seen in table.

-The median age of the entire cohort was 34 years (IQR; 12). More than half (52.1%)

157 of people living with HIV [PLHIV] were females and 323(78.6%) were Orthodox Christian

158 followed by Muslim 60(14.6%) and protestant 28(6.8%) follower

The above statement should be presented in the table

Age group was categorized into :

15-<30;

30-<34

40-<50

≥50

Please state the rationale behind this classification. I do not see the age group of 35 to 30. Could you make clear about this?

-Clinical and treatment-related characteristics should be presented in table form

-Do the patients have any underlying medical conditions? If yes, please present them in table

- -it would be worth presenting baseline Hb level	and prevalence of anemia.

- Some HAART regimens may contribute to developing anemia. Please consider this for analyses.

- Study participants were followed for a minimum of 0.67 and a maximum of 60.63 months.

-What does a minimum of 0.67 mean. Is this a good period for assessing the incidence of anemia?

- Please state the variables that were adjusted in the multivariable Cox regression model.

-This noticeable discrepancy might be related to early initiation of ART for participants in the current study irrespective of CD4 count and WHO clinical staging (**17**) and availability of new drug regimen (dolutegravir containing regimen).

The above statement needs to be justified by the results shown in the study. There is a need to describe the proportion of individuals who received ART, and need to be justified by the available evidence

-Increased serum creatinine related to decrement of renal function to filter it which can result blunt erythropoietin production in response to lower hemoglobin concentration (**30**). This finding suggests that, the need of erythropoietin treatment beside regular monitoring of hemoglobin concentration for patients who have elevated serum

-It is not clear whether patients have had some kind of renal impairment; and this should be clearly mentioned in inclusion/ exclusion criteria.

-
