# Supplementary material for: Incidence and predictors of anemia among adults on HIV care at South Gondar Zone Public General Hospital Northwest Ethiopia, 2020; retrospective cohort study
Source: PLoS One. 2022 Jan 12;17(1):e0259944. doi: 10.1371/journal.pone.0259944 (PMC8754319; doi:10.1371/journal.pone.0259944)
Supplement: Attachment — Submitted filename: 2nd revision response to reviewer comments of Anemia.docx [file pone.0259944.s005.docx]

Agimasie Tigabu, BSc, MSc

Lecturer of Adult Health Nursing, Department of Nursing,

College of Medicine and Health Science

Debre Tabor University,

Debre Tabor, ETHIOPIA

Email: [ethiomom23@gmail.com](mailto:ethiomom23@gmail.com)

Dr. Kwasi Torpey, MD PhD, MPH.

Academic Editor, PLOS ONE

**Aug 21 2020 2:32AM**

My colleagues and I are happy to re-submit to you a revised version of our manuscript PONE-D- 20-26223R1, Incidence and Predictors of Anemia among Adults on HIV care: A Retrospective cohort study. For PLOS One, Thank you for giving us the opportunity to revise and resubmit this manuscript. I am resubmitting this revision on15th October 2021.

We thank the reviewers for their detailed and thoughtful suggestions. We have incorporated the recommended changes in the manuscript to the best of our ability and we feel that our revised manuscript is significantly improved as a result of your advice.

We have responded specifically to each suggestion in the memo below and have highlighted our changes in the manuscript text. In the memo, portions of the reviewers’ comments are reproduced in italic text, and our responses to the reviewers' comments are listed beneath in the Norman text.

The authors received no specific funding for this work.

Thank you once again for your time and attention to our manuscript. We look forward to working with you and the reviewers to move this manuscript closer to publication.

Best regards,

Agimasie Tigabu

REVIEWER COMMENTS AND RESPONSES

**Reviewer's Responses to Questions**

**Comments to the Author**

*1. If the authors have adequately addressed your comments raised in a previous round of review and you feel that this manuscript is now acceptable for publication, you may indicate that here to bypass the “Comments to the Author” section, enter your conflict of interest statement in the “Confidential to Editor” section, and submit your "Accept" recommendation.*

*Reviewer #1: (No Response)*

*Reviewer #2: All comments have been addressed*

Thank you for your suggestion. Actually, we didn’t get such comments if there will be, we will indicate our conflict of interest statement to bypass comments to Author section and enter it to Editor Section. After we got acceptance, we will submit the acceptance recommendation.

We thank you reviewer #2 for your detail understanding of the response for previous round of review comments.

*2. Is the manuscripts technically sound, and do the data support the conclusions?
The manuscript must describe a technically sound piece of scientific research with data that supports the conclusions. Experiments must have been conducted rigorously, with appropriate controls, replication, and sample sizes. The conclusions must be drawn appropriately based on the data presented.*

*Reviewer #1: Partly*

*Reviewer #2: Yes*

Thank you so much for this oversight. As Reviewers said, the manuscript is a scientific research with the data that supports the conclusion and the conclusion has drawn based on the data presented.

*3. Has the statistical analysis been performed appropriately and rigorously?*

*Reviewer #1: Yes*

*Reviewer #2: Yes*

Thank you so much for this suggestion. As the reviewers said, the statistical analysis has been performed appropriately and rigorously.

*4. Have the authors made all data underlying the findings in their manuscript fully available?

The* [*PLOS Data policy*](http://www.plosone.org/static/policies.action#sharing) *requires authors to make all data underlying the findings described in their manuscript fully available without restriction, with rare exception (please refer to the Data Availability Statement in the manuscript PDF file). The data should be provided as part of the manuscript or its supporting information, or deposited to a public repository. For example, in addition to summary statistics, the data points behind means, medians and variance measures should be available. If there are restrictions on publicly sharing data—e.g. participant privacy or use of data from a third party—those must be specified.*

*Reviewer #1: Yes*

*Reviewer #2: Yes*

Thank you so much for this suggestion. All data underlying the findings are fully available within the manuscript.

*5. Is the manuscript presented in an intelligible fashion and written in Standard English?
PLOS ONE does not copyedit accepted manuscripts, so the language in submitted articles must be clear, correct, and unambiguous. Any typographical or grammatical errors should be corrected at revision, so please note any specific errors here.*

*Reviewer #1: No*

*Reviewer #2: Yes*

Thank you so much for this suggestion. The manuscript is written in Standard English.

*6. PLOS authors have the option to publish the peer review history of their article (*[*what does this mean?*](https://journals.plos.org/plosone/s/editorial-and-peer-review-process#loc-peer-review-history)*). If published, this will include your full peer review and any attached files.
If you choose “no”, your identity will remain anonymous but your review may still be made public.*
***Do you want your identity to be public for this peer review?*** *For information about this choice, including consent withdrawal, please see our* [*Privacy Policy*](https://www.plos.org/privacy-policy)*.*

*Reviewer #1:* ***Yes:*** *Shiv Kumar Sah*

*Reviewer #2:* ***Yes:*** *Oumer Abdu Muhie*

Thank you so much for this suggestion. We don’t want to our identity to be public. Thank you.

**Review Comments to the Author**

***Reviewer #1:***

1. *Abstract*

*Anemia is a major public health problem worldwide which accounts 24.8% of the population. Few cross-sectional studies have been conducted on anemia and human immunodeficiency virus. However, it cannot address the incidence and predictors of anemia among human immunodeficiency virus -infected adults.
-The above statement does not support the relevance of the study, as there is a plethora of studies examining this issue globally as well as in Ethiopia also. Please consider rewriting the above statement again
-Moreover, the estimates presented in the background does not seem relevant at this stage*

*Methods are not adequately described- such as population selection and outcome measures need to be clearly mentioned.
-The authors stated that a ethical clearance was obtained from the Institutional Review Board of Bahir Dar University, and also, we got permission letter to review charts from the concerned bodies in the hospital
-The above statement should be rephrased
-This is a similar confusion that which factors were from bivariate and multivariate analyses, and need to be very clearly described, and conclusion must be drawn from the appropriate findings.*

Thank you so much for your constructive comment. We have rewritten and rephrased the statements per the given comments on page 2, line number 29 to 34.

2. *Background:*

*-Line 63-67 seems irrelevant and exaggerated. It should be concise and problem-oriented.
-Please highlight the current burden of the disease globally, and especially in Ethiopia
-The authors failed to clearly summarize the current scenario of the topic. There seems to address the limitation of the current knowledge in this field, especially in Ethiopia
-The authors have revealed some data on the prevalence of anemia in Ethiopia; however, in this particular issue, providing data on the incidence of anemia in this particular setting is vital
-Line 87-89
The authors have indicated some contributing factors of developing anemia, yet there are many potential factors that contribute to this burden that needs to be considered.
-A strong rationale behind this study in this setting is needed
-Overall, the introduction needs to be fine-tuned to be more clear. The numerous grammatical errors and language needs to be considered*.

Thank you so much for your oversight. We have revised and edited the grammatical as well as language errors the background based on the given comments.

3. *Methods:*

*- In the exclusion criteria, authors have excluded only the pregnant women and anemia at the beginning of the follow-up. How about the other potential factors that may contribute to developing anemia during follow-up?
- Please highlight the core outcome variables and covariates analyzed in the study.
- Authors have assessed the adherence level based on the number of doses. I wonder how did the authors approach this, as the review was retrospective. Moreover, is this a standard tool for the assessment of the adherence level. Need citation and reliability of this tool.
- For the sample size calculation, the authors have used the significant predictors from previous studies. I wonder that there are numerous significant predictors for developing anemia, and which of them the author employed for calculating sample size.
-How the missing data were handled?
-In the methodology section, it is vital to include the clinical criteria for the diagnosis of anemia and its severity.*

Thank you so much for your oversight. We have highlighted the core outcome variable in the operational definition part which was dichotomized into event and censored.

Adherence level: - missing dose is a standard tool for the assessment of the adherence level which is taken from ART intake form. We have stated it on page 5, line number 121 to 122.

On sample size calculation: - as you have mentioned that, we have used three significant predictors from previous studies. Those are nutritional status, past TB history and sex. We used the largest of all which is 434 by using nutritional status whereas by using past TB history and sex were given 190 and 44 respectively.

The missing data was handled by adding 10 % non-response rate and by excluding the study participants chart who have greater than or equal to 5% incompleteness.

4. *Results*

*-data should be presented in a well-fashioned manner. I see there are many results described which were not seen in table.
-The median age of the entire cohort was 34 years (IQR; 12). More than half (52.1%)
157 of people living with HIV [PLHIV] were females and 323(78.6%) were Orthodox Christian
158 followed by Muslim 60(14.6%) and protestant 28(6.8%) follower
The above statement should be presented in the table
Age group was categorized into :
15-<30;
30-<34
40-<50
≥50
Please state the rationale behind this classification. I do not see the age group of 35 to 30. Could you make clear about this?
-Clinical and treatment-related characteristics should be presented in table form
-Do the patients have any underlying medical conditions? If yes, please present them in table
- -it would be worth presenting baseline Hb level and prevalence of anemia.
- Some HAART regimens may contribute to developing anemia. Please consider this for analyses.
- Study participants were followed for a minimum of 0.67 and a maximum of 60.63 months.
-What does a minimum of 0.67 mean? Is this a good period for assessing the incidence of anemia?
- Please state the variables that were adjusted in the multivariable Cox regression model.
-Increased serum creatinine related to decrement of renal function to filter it which can result blunt erythropoietin production in response to lower hemoglobin concentration (30). This finding suggests that, the need of erythropoietin treatment besides regular monitoring of hemoglobin concentration for patients who have elevated serum
-It is not clear whether patients have had some kind of renal impairment; and this should be clearly mentioned in inclusion/ exclusion criteria.*

Thank you so much for your constrictive comments. We described the frequencies of some variables in statement way and some are presented in table form.

Age categories: - we categorized it based on previous available literatures. 30 to 34 were written mistakenly. We have categorized it 30 years to 40 years on page 7.

Prevalence of anemia is not the study objective rather we have determined anemia incidence density rate and identified its predictors including their survival probability. We have written it on page 9, line number 185 to 187.

We have studied HAART regimens including changed regimen by considering a predictor variable for developing of anemia. In bivariable cox regression analysis, those regimens were not eligible for multivariable cox regression.

We have followed the study participants for five years duration. during the study period one study participant might be followed for a minimum of 0.67 months and one study participant might be followed for a maximum of 60.63 months.

We have listed the variables which are adjusted in the multivariable Cox regression model on page 11, line number 215 to 217.

Based on the adjusted cox regression analysis result, study participants who have increased serum creatinine level have high risk to develop anemia as compared to who have normal serum creatinine level. Based on this finding, we have written implication for it. Whether the study participants have renal impairment or not if they have fulfilled the inclusion criteria are considered as the study participants.

***Reviewer #2:***

*Thank you for addressing my concerns in the manuscript you submitted to the journal PLOS ONE. I appreciate your effort in that*.

Thank you so much.
